# Salivary microRNA profiling dysregulation in autism spectrum disorder: A pilot study

**DOI:** 10.3389/fnins.2022.945278

**Published:** 2022-10-19

**Authors:** Zamira Kalemaj, Maria Michela Marino, Annamaria Chiara Santini, Giovanni Tomaselli, Amogh Auti, Maria Grazia Cagetti, Tiziana Borsello, Antonella Costantino, Francesco Inchingolo, Mariarosaria Boccellino, Marina Di Domenico, Gianluca Martino Tartaglia

**Affiliations:** ^1^UOC Maxillo-Facial Surgery and Dentistry, Fondazione IRCCS Cà Granda, Ospedale Maggiore Policlinico, Milan, Italy; ^2^Department of Precision Medicine, Università della Campania “Luigi Vanvitelli”, Naples, Italy; ^3^Adolescents Intensive Care Unit, Centre Hospitalier Édouard Toulouse, Marseille, France; ^4^Pharmacological Research Institute Mario Negri-IRCCS, Milan, Italy; ^5^Department of Pharmacological and Biomolecular Sciences, Università di Milano, Milan, Italy; ^6^Department of Biomedical, Surgical and Dental Science, Università di Milano, Milan, Italy; ^7^Child and Adolescent Neuropsychiatric Unit, Fondazione IRCCS Cà Granda, Ospedale Maggiore Policlinico, Milan, Italy; ^8^Section of Dental Medicine, Department of Interdisciplinary Medicine, Università di Bari “Aldo Moro”, Bari, Italy; ^9^Department of Biology, College of Science and Technology, Temple University, Philadelphia, PA, United States

**Keywords:** autism spectrum disorder (ASD), miRNA, salivary poly-omic RNA, biomarkers, miRNAs targeted genes

## Abstract

**Introduction:**

Autism spectrum disorders (ASD) are the most prevalent neurobiological disorders in children. The etiology comprises genetic, epigenetic, and environmental factors such as dysfunction of the immune system. Epigenetic mechanisms are mainly represented by DNA methylation, histone modifications, and microRNAs (miRNA). The major explored epigenetic mechanism is mediated by miRNAs which target genes known to be involved in ASD pathogenesis. Salivary poly-omic RNA measurements have been associated with ASD and are helpful to differentiate ASD endophenotypes. This study aims to comprehensively examine miRNA expression in children with ASD and to reveal potential biomarkers and possible disease mechanisms so that they can be used to improve faction between individuals by promoting more personalized therapeutic approaches.

**Materials and methods:**

Saliva samples were collected from 10 subjects: 5 samples of children with ASD and 5 from healthy controls. miRNAs were analyzed using an Illumina Next-Generation-Sequencing (NGS) system.

**Results:**

Preliminary data highlighted the presence of 365 differentially expressed miRNAs. Pathway analysis, molecular function, biological processes, and target genes of 41 dysregulated miRNAs were assessed, of which 20 were upregulated, and 21 were downregulated in children with ASD compared to healthy controls.

**Conclusion:**

The results of this study represent preliminary but promising data, as the identified miRNA pathways could represent useful biomarkers for the early non-invasive diagnosis of ASD.

## Introduction

Autism spectrum disorder (ASD) is a neurological and developmental disorder characterized by altered social interaction, restricted/repetitive behavior, and medical and mental health conditions that result in lifelong functional and social impairments. It is estimated that ASD is associated with intellectual disability in 70% of cases, with seizures in 30% of cases (Minshew and Williams, 2007). Furthermore, ASD leads to alterations in the circadian cycle: it has been reported that people affected by ASD present a reduced total sleep, with an increased sleep latency. Studies have shown a higher frequency or absence of circadian variation of melatonin and cortisol in ASD patients (Tordjman et al., 2015).

Autism spectrum disorder presents a multifactorial etiology, comprising both genetic and environmental factors (Hallmayer et al., 2011; Singh et al., 2013; Sehovic et al., 2020; Wu et al., 2020). A metanalysis published in 2016, including a cohort of 6,413 twin pairs, estimated that the heritability of ASD to be 74–93% (Tick et al., 2016). Similar values were recently reported by Wu et al. (2020), while moderate heritability values (37–38%) were obtained by Hallmayer et al. (2011) in a Californian study (2011). Both genetic and epigenetic factors may be involved, such as histone modification, DNA methylation, and non-coding sequences of RNA (miRNA) (Zhang et al., 2020). Parental age, *in utero* exposure to several substances including pollution and inflammation, and low birth weight, may also increase the risk (Hallmayer et al., 2011; Sehovic et al., 2020).

The diagnosis of ASD, according to DSM-5, is mainly based on the examination of behavioral characteristics. A person must present deficits in three areas of social interaction: social-emotional reciprocity, non-verbal communicative behaviors, and the ability to maintain and understand relationships (Hallmayer et al., 2011; Wu et al., 2020; Qin et al., 2022). Unfortunately, all these characteristics can be interpreted differently, especially in young patients, leading to delays in diagnosis. Objective hallmarks are therefore needed (Hicks et al., 2020).

The number of children with ASD has grown dramatically over the last 2 decades with a current estimated prevalence of approximately 1 in 60 worldwide, an increase of 154% and even higher figures in future (Sehovic et al., 2020; Salloum-Asfar et al., 2021; Qin et al., 2022). Although the etiology of ASD is still mainly unknown, major genetic aberrations have been recognized in only 25–30% of patients with ASD and are mainly related to pathological mechanisms that start during gestation (Wiśniowiecka-Kowalnik and Nowakowska, 2019). Even if ASD could be diagnosed as early as 18 months of age, on average the diagnosis is done at ∼60 months of age (van’t Hof et al., 2021). Furthermore, as early diagnosis of ASD is often unreliable, effective therapy is usually delayed. This might have negative consequences, considering that the effectiveness of therapy often depends on earliness of treatment beginning (Hicks et al., 2020; Salloum-Asfar et al., 2021). Therefore, recent research on early diagnosis of ASD has focused on large screening programs that can be efficient and easy to implement on a population scale (Hicks et al., 2018a).

Macroscopically, among other brain abnormalities observed in patients with ASD, alterations have been described in Broca and Wernicke’s areas, which are associated with language, and in the frontal lobe, superior temporal cortex, parietal cortex, and amygdala, which are strongly implicated in social behaviors (Ha et al., 2015). However, their use in the clinical practice and to monitor treatment is difficult and expensive as magnetic resonance is needed (Squarcina et al., 2021).

Numerous biomarkers have been studied, measured and analyzed in order to identify potential ADS indicators for both early diagnosis and pathogenesis (Ballini et al., 2020; Hicks et al., 2020; Isacco et al., 2021; Salloum-Asfar et al., 2021). ASD presents typical features of synaptic dysfunction and synaptopathy (Wu et al., 2020). Indeed, aberrant overexpression of the postsynaptic density 95 protein (PSD95), a member of the membrane-associated guanylate kinase that promotes synaptic stability in excitatory synapses, has been observed in patients with ASD. This alteration leads to increased dendritic spines and pathologic synaptic hyperconnectivity (Coley and Gao, 2018). Another important feature of synapses that have been found altered in ASD is SHANK (SH and multiple ankyrin repeat domains protein). SHANK proteins are scaffolding proteins that organize intermediate scaffold proteins. Their location is principally in the excitatory synapse, where they allow the synapse to develop and function properly. Alterations in the SHANK gene have been reported as a possible cause and predictive feature of ASD (Wu et al., 2020).

Among others, miRNA have been proposed as plausible new biomarkers with high predictivity. More than 91 miRNAs associated with ASD have been identified. For instance, alterations of miRNA related to cellular respiration have been found in the serum of patients with ASD. Furthermore, miRNA-500a-5p and miRNA-197-5p expressions in the serum have been suggested as useful tools to diagnose ASD in children (Kichukova et al., 2021). Through analysis of miRNAs, genetic correlations were observed between clinically unaffected parents of children with ASD and their siblings (Ozkul et al., 2020). A great number of miRNA are altered in ASD, and several are related to alteration in central nervous system (Salloum-Asfar et al., 2021; Qin et al., 2022). Preclinical and clinical models of ASD have demonstrated up- and down-regulations of several miRNAs. For instance, upregulation of miRNA-29b, miRNA329, miRNA-199b, miRNA-382, miRNA-296, miRNA-221 and miRNA-92 and downregulation of miRNA-146a, miRNA-146b, miRNA-130, miRNA-122a, miRNA-342 and miRNA-409 were described. Among these targets, for example, miRNA-199 is known to be able to regulate BDNF (Brain Derived Neurotrophic Factor), while miRNA-146a and miRNA-146b have been shown to be involved in the aetiopathogenesis of ASD (Urdinguio et al., 2010). Ivanov et al. (2022), developed a highly sensitive “silicon-on-insulator”-based nanosensor (SOI-NS) for the detection of ASD-associated miRNAs with a lower limit of 10^–17^M.

Nowadays, among all body sampling tissues, saliva is gaining a pivotal role as it is extremely easy and non-invasive to collect and monitor. It should be noted that when choosing a body fluid for biomarker measurement, it is important to avoid stress as much as possible, as stressful procedures can alter the production of hormones such as cortisol that influence the secretion of other hormones through feed-sidewards interaction. Urine or blood collection is more stressful than saliva collection and this makes saliva an appropriate fluid for biological sampling in either patients or healthy subjects. Furthermore, its informative value is considered particularly high, as the salivary proteome is estimated to include more than 3,000 expressed proteins and peptides, comprising biomarkers of neurological diseases (Hicks et al., 2018b,^2020^). After the first reports by Hicks and colleagues, several large-scale screening studies have focused on salivary poly-omic RNA measurements to identify children with ASD (Hicks et al., 2018a; Sehovic et al., 2020). The rise of the ASD epidemic and numerous hypothetical pathways to be elucidated both diagnostically and pathogenetically have given impetus to related research, resulting in an enrichment of recent evidence.

The complexity of ASD makes clinical diagnosis difficult, therefore, by identifying the biomarkers associated with ASD severity and combining them with the diagnosis, it is possible to better divide the factions within the spectrum and devise more targeted therapeutic strategies. To date, there are no reliable biomarkers to diagnose ASD or define its severity. This study aims to comprehensively examine miRNA expression in children with ASD and to reveal potential biomarkers and possible disease mechanisms so that they can be used to improve faction between individuals by promoting more personalized therapeutic approaches.

## Materials and methods

### Samples collection

This prospective comparative and study included 10 children aged between 3 and 7 years. The ASD group comprised 4 males and 1 female (mean age 5.6 years) and the non-ASD group, healthy controls, comprised 3 males and 2 females (mean age 5.4 years) (Table 1). Date on birth weight and parental age were retrieved for all participants and reported on Table 2. For approval, written informed consent was obtained from the parents/caregivers of each participant for approval. Exclusion criteria for the ASD participants included global developmental delay (cognitive disability with IQ < 70), g-tube dependence, confirmed chromosomal deletion or duplication, epilepsy, psychiatric diagnosis and premature birth of more than 6 weeks. Exclusion criteria for the non-ASD groups included a family history of ASD in a first degree relative or a chronic medical condition requiring daily medical care by a specialist. Common exclusion criteria among groups included acute illness, sensory disorders such as hearing or visual impairments, upper respiratory tract and oral cavity infections and wards of the state. Saliva samples were collected from all children in a non-fasting state using Oracollect RNA swabs (DNA Genotek; Ottawa, ON, Canada) through a simple, non-invasive process that consists of applying a highly absorbent swab for a few seconds under the base of the tongue. Each sample collected at least 500 μl of saliva. Samples were immediately sent to the laboratory for analysis. The clinical investigation protocol for this study was submitted to the ethical committee of the Policlinico Fondazione Cà Granda Milano Review Board, and approval has been successfully obtained (727_2021).

**TABLE 1:** Demographical and clinical parameters of ASD subjects.

Patient ID	Sex	Age	Comorbidities	ADOS-II* test score	WPPSI-IV** test score
1	F	5.4	None	7	15
2	M	5.7	None	8	17
3	M	6.6	None	8	16
4	M	4.7	None	9	17
5	M	5.8	None	9	18

*ADOS-II test for ASD diagnosis (ranging from 1 to 10). **WPPSI-IV test for cognitive evaluation (ranging from 1 to 19).

**TABLE 2:** Birth weight and parental age.

Participants	Birth weight (gr)	Parental age (years)	
		Mother	Father
1 Healthy	2,650	25	28
2 Healthy	2,975	32	39
3 Healthy	3,640	28	27
4 Healthy	3,365	29	35
5 Healthy	2,897	27	32
1 ADS	2,495	31	33
2 ADS	2,990	28	35
3 ADS	3,760	24	26
4 ADS	3,805	26	25
5 ADS	2,805	25	27

### miRNAs extraction

miRNAs were extracted from saliva samples using NucleoSpin^®^ miRNA Plasma kit (MACHEREY-NAGEL, GmbH & Co. KG, Germany) and quantified by Agilent 2100 Bioanalyzer RNA assay (Agilent Technologies, Santa Clara, CA, USA). The NucleoSpin miRNA Plasma kit isolates total RNA including miRNA from saliva without the need to resort to the cumbersome phenol/chloroform extraction of a time-consuming proteinase digest. The sample material is denatured in Lysis Buffer MLP. The protein is then precipitated by Protein Precipitation Buffer MPP and pelleted by centrifugation. After the removal of protein, the binding conditions for nucleic acids are adjusted by adding isopropanol. Total nucleic acids are bound to the NucleoSpin miRNA Column. The remaining nucleic acids are washed and eluted with minimal amounts of RNase-free water.

### Library preparation and sequencing

QIAseq miRNA library kit (QIAGEN, Hilden, Germany) has been used for library preparation following the manufacturer’s instructions.

RNA samples were quantified and quality tested by Agilent 2100 Bioanalyzer RNA assay. In addition, final libraries were checked with both Qubit 2.0 Fluorometer (Invitrogen, Carlsbad, CA, USA) and Agilent Bioanalyzer DNA assay. The libraries were sequenced on single-end 150 bp mode on NovaSeq 6000 (Illumina, San Diego, CA, USA).

### Bioinformatic analysis

The bioinformatics analysis of small RNA-Seq includes:

• Base calling and demultiplexing. Processing of raw data for format con-version and demultiplexing by Bcl2Fastq1 2.20 version of the Illumina pipe-line.
• The sRNAbench tool, a sRNAtoolbox2,3 tool, gives expression profiling of small RNAs, prediction of novel microRNAs, analysis of isomiRas, genome mapping, and read length statistics.

### Pathways analysis and gene ontology

To better understand the functional role of the 41 selected miRNAs, a pathway prediction analysis was performed. For this purpose, the bioinformatics tool DIANA-mirPath (version 3) (Vlachos et al., 2015) and miRDB (Chen and Wang, 2020) were used. With this computational approach all target genes and related molecular pathways altered by the selected miRNAs were identified by using this computational approach. Furthermore, GO enrich-ment analysis was performed using the tool GO PANTHER version 14.0.^1^ GO PANTHER analysis was conducted for the lists of genes obtained from DIANA-mirPath analyses and using the NPinter tool to evaluate the interactors of the top 10 dysregulated miRNAs.

### Statistical analyses

Fold change values of miRNA expression levels were calculated through differential analysis. Student’s *t*-tests were performed to select the differentially expressed miRNAs with a statistical significance, as reported in Tables 3, 4. For the Kaplan–Meier survival analysis, the non-parametric log-rank test was used to compare the survival distributions of the patients with ASD according to the down-regulation or overexpression of selected miRNAs. Data with a *P*-adj ≤ 0.05 were considered to indicative of statistically significant differences.

**TABLE 3:** miRNAs expression in ASD compared to healthy control.

miRNA	miRNA ID (mirBase)	log2 FC	Adjusted *p*-value	No.
**Upregulated miRNAs**				
hsa-miR-1246	MIMAT0005898	4,793	2.87E-05	5
hsa-miR-199b-5p	MIMAT0000263	3,590	0.00038	5
hsa-miR-4516	MIMAT0019053	3,483	0.00090	5
hsa-miR-199a-3p	MIMAT0000231	3,379	0.00030	5
hsa-miR-199b-3p	MIMAT0000263	3,363	0.00034	5
hsa-miR-223-5p	MIMAT0004570	3,274	3.62E-07	5
hsa-miR-143-3p	MIMAT0000435	3,207	0.00026	5
hsa-miR-223-3p	MIMAT0000280	3,193	0.00026	5
hsa-miR-142-3p	MIMAT0000434	3,013	0.00324	5
hsa-miR-338-3p	MIMAT0000763	2,971	0.00929	5
hsa-miR-145-5p	MIMAT0000437	2,860	0.00237	5
hsa-miR-142-5p	MIMAT0000433	2,847	0.00034	5
hsa-miR-150-5p	MIMAT0000451	2,508	0.01510	5
hsa-miR-146a-5p	MIMAT0000449	2,326	5.86E-06	5
hsa-miR-155-5p	MIMAT0000646	2,189	0.01987	5
hsa-miR-542-3p	MIMAT0003389	2,050	0.00106	5
hsa-miR-424-5p	MIMAT0001341	1,710	0.00219	5
hsa-miR-140-5p	MIMAT0000431	1,529	0.00541	5
hsa-miR-191-5p	MIMAT0000440	1,357	0.00026	5
hsa-miR-628-5p	MIMAT0004809	1,259	0.03283	5
hsa-miR-140-3p	MIMAT0004597	1,185	0.00172	5
**Downregulated miRNAs**				
hsa-miR-545-5p	MIMAT0004785	−2,976	0.01466	1
hsa-miR-660-3p	MIMAT0022711	−2,505	0.03171	1
hsa-miR-4284	MIMAT0016915	−2,284	0.00035	3
hsa-miR-203a-5p	MIMAT0031890	−2,261	0.00221	2
hsa-miR-1973	MIMAT0009448	−2,186	0.00035	4
hsa-miR-545-3p	MIMAT0003165	−1,874	0.03898	3
hsa-miR-1277-3p	MIMAT0005933	−1,737	0.00491	5
hsa-miR-1277-5p	MIMAT0022724	−1,653	3.62E-07	5
hsa-miR-4485-3p	MIMAT0019019	−1,582	0.017670	5
hsa-miR-193a-3p	MIMAT0000459	−1,566	0.018020	3
hsa-miR-33a-5p	MIMAT0000091	−1,459	0.005697	5
hsa-miR-190a-5p	MIMAT0000458	−1,441	0.004909	4
hsa-miR-561-5p	MIMAT0022706	−1,346	0.002212	5
hsa-miR-33b-5p	MIMAT0003301	−1,328	0.038566	5
hsa-miR-1307-5p	MIMAT0022727	−1,324	0.009289	5
hsa-miR-219a-5p	MIMAT0000276	−1,284	0.001110	5
hsa-miR-34a-3p	MIMAT0004557	−1,153	0.014613	5
hsa-miR-378d	MIMAT0018926	−1,139	0.018601	4
hsa-miR-149-5p	MIMAT0000450	−1,122	0.032315	5
hsa-miR-96-5p	MIMAT0000095	−1,017	0.003727	5

**TABLE 4:** Kegg pathway of 21 upregulated miRNAs.

KEGG pathway	*p*-value	No. of genes	#miRNAs
Hippo signaling pathway (hsa04390)	5.48E + 03	79	21
Signaling pathways regulating pluripotency of stem cells (hsa04550)	2.86E + 06	68	21
Thyroid hormone signaling pathway (hsa04919)	3.96E + 06	58	21
Ubiquitin mediated proteolysis (hsa04120)	0.01791	61	21
Proteoglycans in cancer (hsa05205)	5.28E + 04	94	20
Axon guidance (hsa04360)	3.76E + 06	58	20
**Wnt signaling pathway (hsa04310)**	9.89E + 06	68	20
Glioma (hsa05214)	0.0008	32	20
Pancreatic cancer (hsa05212)	0.0008	36	20
FoxO signaling pathway (hsa04068)	0.0018	61	20
Ras signaling pathway (hsa04014)	0.0019	95	20
**PI3K-Akt signaling pathway (hsa04151)**	0.0019	142	20
Pathways in cancer (hsa05200)	0.0020	166	20
Regulation of actin cytoskeleton (hsa04810)	0.0042	97	20
Focal adhesion (hsa04510)	0.0045	91	20
**Neurotrophin signaling pathway (hsa04722)**	0.0053	57	20
Chronic myeloid leukemia (hsa05220)	0.0111	36	20
Oxytocin signaling pathway (hsa04921)	0.0151	66	20
Glutamatergic synapse (hsa04724)	0.0225	50	20
Insulin signaling pathway (hsa04910)	0.0461	60	20
ErbB signaling pathway (hsa04012)	0.0007	46	19
**TGF-beta signaling pathway (hsa04350)**	0.0019	33	19
Adherens junction (hsa04520)	0.0019	39	19
Prostate cancer (hsa05215)	0.0044	45	19
Adrenergic signaling in cardiomyocytes (hsa04261)	0.0142	61	19
Arrhythmogenic right ventricular cardiomyopathy (ARVC) (hsa05412)	0.0161	35	19
Choline metabolism in cancer (hsa05231)	0.0189	48	19
Oocyte meiosis (hsa04114)	0.0191	48	19
Dilated cardiomyopathy (hsa05414)	0.0195	41	19
GABAergic synapse (hsa04727)	0.0200	37	19
Melanoma (hsa05218)	0.0223	34	19
Cholinergic synapse (hsa04725)	0.0461	52	19
Insulin secretion (hsa04911)	0.0470	38	19
T cell receptor signaling pathway (hsa04660)	0.0480	46	19
N-Glycan biosynthesis (hsa00510)	0.0001	23	18
Renal cell carcinoma (hsa05211)	0.0022	37	18
Non-small cell lung cancer (hsa05223)	0.0042	29	18
Colorectal cancer (hsa05210)	0.0046	32	18
**Hedgehog signaling pathway (hsa04340)**	0.0069	28	18
Sphingolipid signaling pathway (hsa04071)	0.0111	53	18
AMPK signaling pathway (hsa04152)	0.0224	55	18
Phosphatidylinositol signaling system (hsa04070)	0.0471	36	18
Endometrial cancer (hsa05213)	0.0042	29	17
Central carbon metabolism in cancer (hsa05230)	0.0179	32	17
Type II diabetes mellitus (hsa04930)	0.0255	24	17
**Long-term depression (hsa04730)**	0.0260	30	17
Lysine degradation (hsa00310)	0.0138	21	16
**Circadian rhythm (hsa04710)**	0.0282	17	16
Dorso-ventral axis formation (hsa04320)	0.0242	16	15
Fatty acid metabolism (hsa01212)	0.0009	20	14
Prion diseases (hsa05020)	5.28E + 04	11	9
Fatty acid biosynthesis (hsa00061)	1.28E-01	6	6

## Results

### Identification of dysregulated miRNAs

**FIGURE 1:**
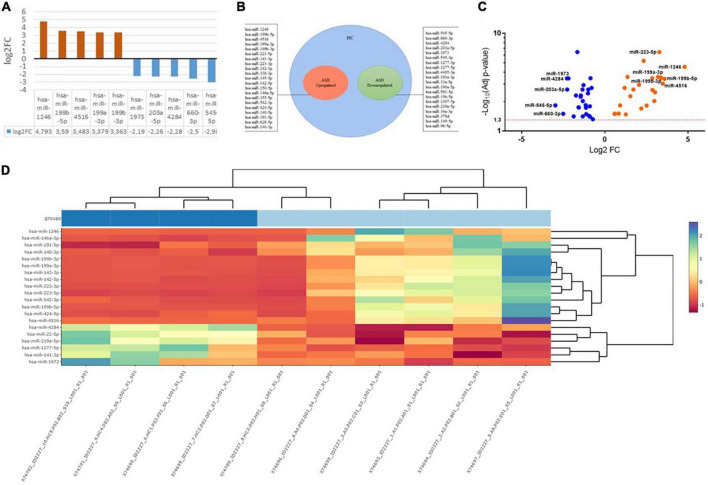
**(A)** The top miRNAs dysregulated in ASD patients compared to healthy control. **(B)** Venn Diagram. **(C)** Volcano plot of the identified miRNAs. **(D)** Heat map diagram showing cluster analysis of differently expressed miRNAs. The miRNAs selected are differentially expressed between ASD and HC (Healthy Control) groups (*p* < 0.05). The color scale reflects the signal strength and goes from green (low intensity) to black (medium intensity) to red (high intensity).

Saliva samples were collected from 10 subjects: 5 samples from children with ASD and 5 from healthy controls, respectively. Preliminary data highlighted the presence of 365 differentially expressed miRNAs. Applying a more stringent statistical analysis (*p*adj < 0.05) to create hierarchical clustering, we found 41 dysregulated miRNAs were found, of which 20 were upregulated and 21 miRNAs were downregulated in saliva from children with ASD compared to from healthy controls (Table 3). Figure 1 shows the top five upregulated miRNAs in ASD patients compared to healthy control. Venn diagram, Volcano plot of the identified miRNAs, and heat map diagram showing cluster analysis of differently expressed miRNAs: hsa-miRNA-1246 (5-fold, *p* < 0.00000035), hsa-miRNA-199b-5p (3.59-fold, *p* < 0.000014), hsa-miRNA-4516 (3.5-fold, *p* < 0.000004), hsa-miRNA-199a-3p (3.4-fold, *p* < 0.000006), hsa-miRNA-199b-3p (3.36-fold, *p* < 0.000008), and the top five downregulated: hsa-miRNA-545-5p (3-fold, *p* < 0,001), hsa-miRNA-660-3p (2.5-fold, *p* < 0.000006), hsa-miRNA-4284 (2.28-fold, *p* < 0.000006), hsa-miRNA-203a-5p (2.26-fold, *p* < 0.0001), hsa-miRNA-1973 (2.2-fold, *p* < 0.000006). The miRNAs selected are differentially expressed between ASD and HC (Healthy Control) groups (*p* < 0.05).

### Identification of the miRNAs targeted genes and correlation analysis

**FIGURE 2:**
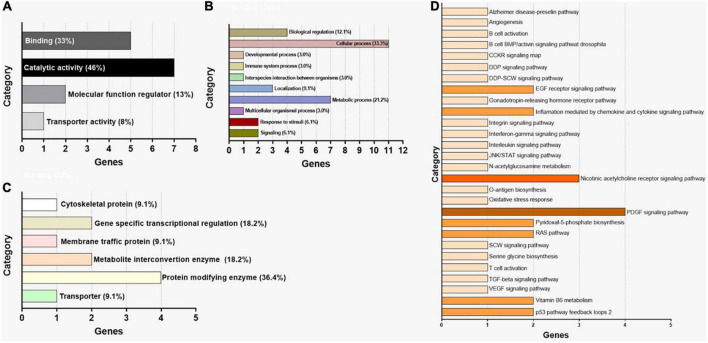
Gene ontology enrichment analysis by PANTHER for the 15 genes identified through miEEA. **(A)** Distribution of genes according to molecular function. **(B)** Distribution of genes according to biological process. **(C)** Distribution of genes according to protein class. **(D)** Distribution of genes according to signaling pathway.

For the pathway prediction analysis, all the 41 miRNAs were entered into the search bar of DIANA mirPath. The analysis revealed that modulated pathways and target genes existed for these miRNAs according to TarBase version 7.0 the reference database of DIANA mirPath. By performing a differential analysis of all the molecular pathways altered by miRNAs up- and downregulated in ASD, it was possible to establish that all reported miRNAs could modulate several biological and pathological pathways, as reported in Tables 4, 5. In detail, up and downregulated miRNAs in ASD can alter 53 and 50 different pathways, respectively. Among the altered pathways, 11 were directly related to ASD. Among these 11 pathways, the most affected pathways were the Wnt signaling pathway and Axon Guidance (20 miRNAs up-regulated and 16 downregulated), PI3K-Akt signaling pathway (20 up- and15 downregulated), GABAergic synapse and TGF-beta signaling pathway (19 up- and 14 downregulated), Jak-STAT signaling pathway (19 miRNAs downregulated), circadian entrainment (13 miRNAs downregulated) and circadian rhythm (16 upregulated), Prion diseases (9 upregulated and 9 downregulated), Hedgehog signaling pathway (only 18 upregulated), Neurotrophin signaling pathway (only 20 miRNAs upregulated). Moreover, Hippo pathway, which is regulated by all 21 upregulated miRNAs and 16 downregulated miRNAs, is involved in neurodegenerative diseases. Therefore, it is evident that a high number of selected miRNAs were involved in the development of ASD and neurodegeneration (miRTarBase) (Tables 4, 5). Subsequently, all genes related to each miRNA were merged. In total, 12 genes were identified in common among all the 41 selected miRNAs were identified using this approach (mirPATHv.3). As shown in Table 6, the target genes of the 41 dysregulated miRNAs are PSAT1, STAT1, PIM1, MYO6, USP48, TWSG1, IL6, RBBP9, MMP14, ARL15, TRPC4, BRAF (Table 6). Using the same criteria, Table 7 lists only the top 10 dysregulated miRNAs; target genes are ZNF106, GFPT2, JUNB, VPS33A. The subsequent analysis evaluated the interactors of the miRNAs. Twenty-three interactors were revealed by the analysis of the top 10 dysregulated miRNAs (Table 8) (NPinter). Involved in ASD were: AASDHPPT (hsa-miRNA-1246; hsa-miRNA-199b-5p; hsa-miRNA-199a-3p; hsa-miRNA-199b-3p); AUTS2 (hsa-miRNA-199b-5p; hsa-miRNA-199a-3p; hsa-miRNA-199b-3p). IRDF1 is a predicted gene in ASD (hsa-miRNA-199b-5p; hsa-miRNA-199a-3p; hsa-miRNA-199b-3p) (Table 8). The last step of the analysis consisted of the GO enrichment analysis by PANTHER, listing the 15 genes. As shown in Figure 2, the selected genes were grouped according to molecular function, biological process, and protein class (Figure 2). As regards the molecular function, it was observed that the majority of genes were involved in catalytic activity (GO:0003824) functions (46% of genes) (Figure 2A). When considering the biological processes, 68.8–43.8% of the genes were involved in cellular processes (GO:0009987) and metabolic processes (GO:0008152) (Figure 2B). The most represented protein classes were protein modifying enzyme (PC00260), transporter (PC00227), and metabolite interconversion enzyme (Figure 2C). Finally, pathway analysis revealed the association to molecular mechanisms for only a few genes. Indeed, only 4 genes out of the 15 recognized were assigned to a molecular pathway (Figure 2D). The most represented pathways were the Inflammation mediated by chemokine and cytokine signaling pathway (P00031), Interleukin signaling pathway (P00036), PDGF signaling pathway (P00047), Alzheimer’s disease-presenilin pathway (P00004), and EGF receptor signaling pathway (P00018).

**TABLE 5:** KEGG pathway 20 downregulated miRNAs.

KEGG pathway	*p*-value	No. of genes	No. of miRNAs
Pathways in cancer (hsa05200)	6.15E + 05	175	18
Endocytosis (hsa04144)	0.00134	90	17
**Hippo signaling pathway (hsa04390)**	0.00025	64	16
Ras signaling pathway (hsa04014)	0.00064	94	16
Transcriptional mis regulation in cancer (hsa05202)	0.00148	79	16
ErbB signaling pathway (hsa04012)	0.00745	41	16
Axon guidance (hsa04360)	0.00745	59	16
Wnt signaling pathway (hsa04310)	0.04625	56	16
Proteoglycans in cancer (hsa05205)	1.80E + 04	92	15
PI3K-Akt signaling pathway (hsa04151)	1.47E + 06	148	15
Morphine addiction (hsa05032)	3.38E + 06	41	15
Renal cell carcinoma (hsa05211)	6.32E + 06	36	15
FoxO signaling pathway (hsa04068)	8.37E + 06	64	15
Chronic myeloid leukemia (hsa05220)	0.00024	39	15
Prostate cancer (hsa05215)	0.00074	45	15
Acute myeloid leukemia (hsa05221)	0.00162	30	15
Regulation of actin cytoskeleton (hsa04810)	0.00423	90	15
Colorectal cancer (hsa05210)	0.00745	31	15
Pancreatic cancer (hsa05212)	0.00745	32	15
Ubiquitin mediated proteolysis (hsa04120)	0.01635	56	15
Jak-STAT signaling pathway (hsa04630)	0.01635	66	15
MAPK signaling pathway (hsa04010)	0.03528	99	15
Oxytocin signaling pathway (hsa04921)	0.04960	63	15
GABAergic synapse (hsa04727)	3.27E + 05	38	14
Glioma (hsa05214)	1.65E + 06	34	14
TGF-beta signaling pathway (hsa04350)	5.38E + 06	39	14
Melanoma (hsa05218)	5.38E + 06	40	14
Choline metabolism in cancer (hsa05231)	0.00032	51	14
Thyroid hormone signaling pathway (hsa04919)	0.00148	52	14
Rap1 signaling pathway (hsa04015)	0.00423	90	14
Phosphatidylinositol signaling system (hsa04070)	0.00505	36	14
Estrogen signaling pathway (hsa04915)	0.01385	42	14
Cholinergic synapse (hsa04725)	0.03249	48	14
cAMP signaling pathway (hsa04024)	0.04960	79	14
Signaling pathways regulating pluripotency of stem cells (hsa04550)	0.00027	64	13
Prolactin signaling pathway (hsa04917)	0.00423	34	13
Lysine degradation (hsa00310)	0.00505	20	13
Circadian entrainment (hsa04713)	0.01663	44	13
mTOR signaling pathway (hsa04150)	0.04513	28	13
Endometrial cancer (hsa05213)	0.03739	26	12
Thyroid cancer (hsa05216)	0.04513	13	12
Non-small cell lung cancer (hsa05223)	0.04513	25	12
HIF-1 signaling pathway (hsa04066)	0.04867	44	12
Bacterial invasion of epithelial cells (hsa05100)	0.04960	32	12
Prion diseases (hsa05020)	1.54E + 02	14	9
Dorso-ventral axis formation (hsa04320)	0.045132	15	9
Mucin type O-Glycan biosynthesis (hsa00512)	9.65E + 04	14	8
Fatty acid biosynthesis (hsa00061)	2.11E-01	6	4
Glycosaminoglycan biosynthesis–chondroitin sulfate/dermatan sulfate (hsa00532)	0.03317	7	3

**TABLE 6:** Target gene of 41 dysregulated miRNAs.

Target genes	*P*-value	*P*-adjusted	miRNAs
PSAT1	1.98e-5	0.016	8
STAT1	2.68e-7	0.0013	6
PIM1	4.53e-5	0.029	6
MYO6	5.19e-5	0.029	6
USP48	1.09e-4	0.049	6
TWSG1	3.14e-6	0.008	5
IL6	1.76e-5	0.0169	5
RBBP9	4.20e-5	0.0291	5
MMP14	1.72e-5	0.0169	4
ARL15	1.16e-4	0.049	4
TRPC4	1.51e-5	0.0169	3
BRAF	7.36e-5	0.0378	3

**TABLE 7:** Target gene of enrichment analysis of 10 dysregulated miRNAs.

Subcategory	*P*-value	*P*-adjusted	miRNAs
ZNF106	1.18e-4	0.0496188	4
GFPT2	7.29e-5	0.0496188	3
JUNB	1.19e-4	0.0496188	3
VPS33A	1.33e-4	0.0496188	3

**TABLE 8:** Interactors of top 10 miRNAs dysregulated.

Interactor	*P*-value	*P*-adjusted	Observed	miRNAs/precursors
LMO3	4.34e-4	0.0408158	4	hsa-miR-199b-5p; hsa-miR-199a-3p; hsa-miR-199b-3p; hsa-miR-4284
AASDHPPT	0.0019694	0.0496852	4	hsa-miR-1246; hsa-miR-199b-5p; hsa-miR-199a-3p; hsa-miR-199b-3p
VEGFA	0.0018716	0.0496852	4	hsa-miR-1246; hsa-miR-199b-5p; hsa-miR-199a-3p; hsa-miR-199b-3p
IFRD1	1.77e-5	0.0094881	3	hsa-miR-199b-5p; hsa-miR-199a-3p; hsa-miR-199b-3p
ZNF763	1.77e-5	0.0094881	3	hsa-miR-1246; hsa-miR-199a-3p; hsa-miR-199b-3p
FGFR1OP2	9.68e-5	0.0259363	3	hsa-miR-199a-3p; hsa-miR-199b-3p; hsa-miR-4284
SEC61B	8.48e-5	0.0259363	3	hsa-miR-1246; hsa-miR-199a-3p; hsa-miR-199b-3p
TRIM71	7.39e-5	0.0259363	3	hsa-miR-199a-3p; hsa-miR-199b-3p; hsa-miR-4284
AMZ2	5.71e-4	0.0408158	3	hsa-miR-199b-5p; hsa-miR-199a-3p; hsa-miR-199b-3p
EHD3	4.26e-4	0.0408158	3	hsa-miR-1246; hsa-miR-199a-3p; hsa-miR-199b-3p
MBD1	5.32e-4	0.0408158	3	hsa-miR-199b-5p; hsa-miR-199a-3p; hsa-miR-199b-3p
TBC1D4	4.26e-4	0.0408158	3	hsa-miR-1246; hsa-miR-199a-3p; hsa-miR-199b-3p
WIPI2	6.99e-4	0.0408158	3	hsa-miR-199b-5p; hsa-miR-199a-3p; hsa-miR-199b-3p
AP2A2	8.44e-4	0.0437519	3	hsa-miR-199a-3p; hsa-miR-199b-3p; hsa-miR-4284
MAP1A	8.96e-4	0.0437519	3	hsa-miR-199a-3p; hsa-miR-199b-3p; hsa-miR-4284
MOG	0.0011897	0.0443354	3	hsa-miR-199a-3p; hsa-miR-199b-3p; hsa-miR-4284
RPL37A	0.0010074	0.0443354	3	hsa-miR-199b-5p; hsa-miR-199a-3p; hsa-miR-199b-3p
USP33	0.0012549	0.0443354	3	hsa-miR-199a-3p; hsa-miR-199b-3p; hsa-miR-4284
ZDHHC21	0.0010074	0.0443354	3	hsa-miR-1246; hsa-miR-199a-3p; hsa-miR-199b-3p
ZNF227	0.0011267	0.0443354	3	hsa-miR-1246; hsa-miR-199a-3p; hsa-miR-199b-3p
AUTS2	0.0019469	0.0496852	3	hsa-miR-199b-5p; hsa-miR-199a-3p; hsa-miR-199b-3p
LIMD2	0.0015385	0.0496852	3	hsa-miR-199b-5p; hsa-miR-199a-3p; hsa-miR-199b-3p
PCYOX1	0.0018602	0.0496852	3	hsa-miR-199a-3p; hsa-miR-199b-3p; hsa-miR-4284
SCARNA9	0.0021278	0.0496852	3	hsa-miR-199b-5p; hsa-miR-199a-3p; hsa-miR-199b-3p

## Discussion

Autism spectrum disorders is a highly heritable neurodevelopmental disease with a sharply increasing incidence (Santini et al., 2014). The diagnosis of ASD is mainly based on psychometric evaluations and is often difficult and subjective. Therefore, molecular detection for an early and objective ASD diagnosis is indispensable. This study is focused on the non-invasive evaluation of miRNA/gene biomarkers collected from salivary samples of children. As aforementioned, miRNAs are small non-coding regulatory RNAs that play an essential role in fundamental neurobiological processes and neurodevelopmental diseases. From preliminary data, dysregulated miRNAs in ASD are presented in several pathways. As expected in the canonical Wnt pathway, axon guidance and GABAergic synapse pathways adversely affect neurodevelopment and lead to the pathogenesis of ASD (Giudice et al., 2017; Somekh, 2021). The data obtained show that many miRNAs are involved in the TGF-beta signaling pathway (Boccellino et al., 2017; Dana et al., 2020), involved in ASD development, justifying the presence of dysregulated miRNAs in these other pathways. The present findings show that the adherents junctions, FoxO, Hippo and the Hedgehog signaling pathway are altered.

Interestingly, miRNA-199b-5p increased significantly in saliva of children with ASD, which is involved in the regulation of the AUTS2 gene («miRDB» database). The miRNA-199a-3p, which was observed to be strongly increased in the present work, is considered to be involved in the regulation, among all the other targets, of the CB1 receptor (Cannabinoid receptor type 1) («miRDB» database). Indeed, CB1 receptor, responsible for the social reward, has been observed reduced in the brain of people with ASD postmortem (Shohami et al., 2011). The miRNA-4516 was also found upregulated in the serum of ASD patients (Huang et al., 2015), which is considered to be involved in the sodium voltage-gated channel («miRDB» database). Altered gene expression of this channel is considered to be a solid predictor of ASD (Yamakawa, 2016). As previously observed in the blood of children with ASD (Huang et al., 2015), an increase of miRNA-1246 in saliva was also found. miRNA-1246 is predicted to be implicated in the regulation of SYN II (Synapsin II) («miRDB» database), which was identified as an ASD predisposition gene (Corradi et al., 2014). Also, miRNA-1246 together with the miRNA-4516, is involved in circadian cycles, and metabolic processes («miRDB» database). Furthermore, miRNA-1246 is predicted to regulate the expression of the voltage-gated sodium channel («miRDB» database). In this sense, the discovery of an altered miRNA-1246 in the saliva, which follows the same trend as observed in the peripheral blood, suggests that it could be a suitable peripheral, non-invasive biomarker for the early diagnosis of ASD.

Furthermore, a decrease in miRNA-454-5p, which is among the other functions is involved in CREB (cAMP Response Element-Binding) regulation («miRDB» database) was observed. miRNA-660-5p was also found decreased in saliva of children with ASD. Interestingly, this miRNA is implicated in regulating insulin receptor substrate 1 («miRDB» database). miR-4284 was found to be decreased in patients with ASD. This miRNA is in fact implicated, as is miR-1246, in the regulation of SYN II gene («miRDB» database). Miao et al. (2021), identified miR-4284 as an anticancer in colon cancer. It inhibits colon cancer tumorigenesis by reducing PLIN5 and inhibiting EMT. Thus, miR-4284 could be a potential therapeutic target in metastatic colon cancer. Another miRNA that resulted downregulated is miRNA-203a-5p. This miRNA is predicted to be implicated in the regulation of ERK1 («miRDB» database) (Vanacore et al., 2017; Cossu et al., 2019). Lastly, a decrease of the miR-1973 was observed. This miRNA is associated, among other functions, with the regulation of the potassium gated voltage channel.

Furthermore, analyses performed on the genes regulated by the first 5 up- and downregulated miRNAs showed the main pathways related to these miRNAs. Among other pathways, it was found that the EGF (epidermal growth factor) receptor signaling was affected by two miRNAs. Consistent with the present findings, the EGFR signaling was observed to be altered in children with ASD (Vallés and Barrantes, 2021). Another interesting pathway observed is that of the nicotinic receptor’s pathway, which resulted regulated by three different miRNAs. Indeed this receptor was observed to be altered in people with ASD, and more in particular in the frontal and parietal cortex, as well as in the cerebellum, which is deeply implied in social cognition, which means that cerebellar alteration could be involved in eye avoidance and similar behaviors. PDGF (Platelet-Derived Growth Factor), was the single gene most regulated by the miRNA studied in the present work, with a total of four different miRNAs. Although there are only a few papers are available that demonstrate a connection between PDGF and ASD, it should be noted that PDGF was indeed observed increased in the plasma of children with ASD (Zakareia et al., 2012). Another noteworthy element that was found regulated by a miRNA from the studied pool, was the JAK/STAT signaling. The tumor suppressor p53 was also observed regulated by two different miRNAs of the first five analyzed. In fact, in a preclinical model of ASD, it was suggested that dysregulation of p53 was correlated with altered DNA repair capacity and genomic instability that might lead to ASD (Wong et al., 2016). Some of the miRNAs in our study are found to be associated with inflammatory and proliferative diseases (Table 9). In particular, hsa-miRNA-1246 has been reported as a biomarker secreted in different biological samples in many neoplastic diseases. In fact, studies in colorectal cancer cell lines have shown oncogenic role of miR-1246. In this cancer, the methyltransferase METTL3 oncogene has been shown to increase methylation of pri-miR-1246 to enhance maturation of pri-miR-1246. Notably, miR-1246 has been predicted to suppress expression of the Sprouty Related EVH1 Domain Containing 2 (SPRED2) tumor suppressor, by increasing MAPK pathway (Peng et al., 2019). In this study, we would like to report that this miRNA is upregulated with highest fold change.

**TABLE 9:** Reports of disease association for the dysregulated miRNAs in current study.

miRNA	Biological sample	Disease	References
hsa-mir-1246	Plasma	Systemic lupus erythematosus	Ishibe et al., 2018
hsa-mir-1246	Whole blood	Heart failure	Nair et al., 2013
hsa-mir-1246	Serum	Esophageal neoplasms	Takeshita et al., 2013
hsa-mir-1246	Serum	Early-stage cervical squamous cell carcinoma	Chen et al., 2013
hsa-mir-1246	Plasma	Melanoma	Armand-Labit et al., 2016
hsa-mir-1246	Serum	Acute myeloid leukemia	Hornick et al., 2015
hsa-mir-1246	Serum	Breast neoplasms	Shimomura et al., 2016
hsa-mir-1246	Serum	Carcinoma, ovarian, serous	Todeschini et al., 2017
hsa-mir-1246	Plasma exosome	Pancreatic neoplasms	Xu et al., 2017
hsa-mir-1246	PBMC	Dengue virus infection	Jiang and Sun, 2018
hsa-mir-1246	Serum	Colorectal carcinoma	Guo et al., 2018
hsa-mir-1246	Whole blood	Plasmodium falciparum malaria	Li et al., 2018
hsa-mir-1246	SiHa cells	Cervical carcinoma	Du et al., 2018
hsa-mir-1246	Colorectal tumors	Colorectal carcinoma	Piepoli et al., 2012
hsa-mir-1246	HepG2 cells	Hepatocellular carcinoma	Yan et al., 2013
hsa-mir-1246	Whole blood	Hemophilia A	Sarachana et al., 2015
hsa-mir-1246	HCC1588 cells	Non-small-cell lung carcinoma	Kim et al., 2016
hsa-mir-1246	Saliva	Biliary tract carcinoma	Machida et al., 2016
hsa-mir-1246	SiHa cervical cancer cell	Cerevial squamous cell carcinoma	Chen et al., 2014
hsa-mir-1246	Exosomes	Alcoholic hepatitis	Momen-Heravi et al., 2015
hsa-mir-1246	HCC cells	Hepatocellular carcinoma	Sun et al., 2014
hsa-mir-1246	Panc1 cells	Pancreatic neoplasms	Hasegawa et al., 2014
hsa-mir-1246	Serum Exosomes	Pancreatic neoplasms	Madhavan et al., 2015
hsa-mir-1246	BC tumor tissue	Breast carcinoma	Chen et al., 2016
hsa-mir-1246	Serum exosome	Gastrointestinal neoplasms	Wei et al., 2018
hsa-mir-1246	Plasma exosome	Breast carcinoma	Zhai et al., 2018
hsa-mir-1973	K562 cell-derived exosomes	Chronic myeloid leukemia	Feng et al., 2013
hsa-mir-1973	Serum	Hodgkin lymphoma	Jones et al., 2014
hsa-mir-378d	Serum	Lung neoplasms	He et al., 2018
hsa-mir-4284	Colonic tissue	Ulcerative colitis	Koukos et al., 2015
hsa-mir-4284	GBM specimen	Glioblastoma	Yang et al., 2014

Furthermore, our analysis showed that genes in significant pathways that are predicted to be targeted by deregulated miRNAs control apoptosis, cell death of immune cells, cell cycle progression, epilepsy and neuronal cell proliferation. Our results reinforce the findings of other groups regarding important pathways, such as the regulation of cell growth, the role of organs besides the brain in ASD, and overlap with other non-neurodevelopmental diseases, such as cancer.

## Conclusion

In our pilot study, we screened differentially expressed miRNAs in the saliva sample of ASD children and healthy control. Our sequencing investigation identified the significant number of miRNAs that were up-or down-regulated in the ASD group. The targets of miRNAs under study have been observed through specific tools to predict associated pathways and its regulation. The dysregulated miRNAs and their target genes were found to be involved in developmental pathways already known as the cause of typical ASD and neurobiological dysfunctions. This pilot study can be improved on biostatistical grounds by increasing the size of ASD cohort. Also, the lack of proteomic and genetic validation by RT-PCR of the specific miRNAs, and target genes, remains an unfinished task to claim these miRNAs as biomarkers for ASD.

## Data availability statement

The raw data supporting the conclusions of this article will be made available by the authors, without undue reservation.

## Ethics statement

The study was conducted in accordance with the Declaration of Helsinki and approved by Ethics Committee “DIAGNOSI PRECOCE DEI DISTURBI DELLO SVILUPPO NEUROLOGICO IN SALIVA, INDIVIDUAZIONE DI MARCATORI PREDITTIVI ATTRAVERSO BIOSENSORI” Fondazione IRCCS Ca’ Granda Ospedale Maggiore Policlinico, Milano, U.O.C. Chirurgia Maxillo-Facciale e Odontostomatologia (727_2021). Written informed consent to participate in this study was provided by the participants’ legal guardian/next of kin.

## Author contributions

ZK, MM, AS, and GTo participated in the conceptualization and writing-original draft preparation. MM and GTo participated in the methodology and software. AA participated in the data curation. MC and TB participated in the formal analysis. AC and FI participated in the resources. MB, MD, and GTa participated in the validation, supervision, and writing-review and editing. All authors have read and agreed to the published version of the manuscript.

## Footnotes

1
http://pantherdb.org/
